# Appendicitis—Some Remarks on Its Pathology, Diagnosis and Treatment*Read at Galveston meeting South Texas Medical Association, December 9, 1903.

**Published:** 1904-01

**Authors:** F. L. Barnes

**Affiliations:** Trinity, Texas


For the Texas Medical Journal.

### Appendicitis—Some Remarks on its Pathology, Diagnosis and Treatment.*

F. L. BARNES, M. D., TRINITY, TEXAS.

The pathological conditions that may be found in and around a diseased appendix are many and varied. The following classifications and subdivisions are based upon these pathological findings:

(1) Acute Appendicitis
- (a) Acute catarrhal.
- (b) Acute perforating with circumscribed peritonitis.
- (c) Acute perforating with diffuse septic peritonitis.

(2) Sub acute Appendicitis.

(3) Chronic Appendicitis

- (a) Apendicitis obliterans.

- (b) Appendicitis recurrens or relapsing.

Acute appendicitis may or may not involve the walls of the appendix; there may be simply a catarrhal inflammation of the mucous membrane lining the appendix, or the membrane at certain points may ulcerate, and this ulceration may extend more or less deeply into the walls of the appendix. This variety of the disease may end in complete recovery, or progress into one of the

*Read at Galveston meeting South Texas Medical Association, December 9, 1903.

other varieties of appendicitis. The appendix in these cases is surrounded by plastic inflammation.

In acute perforating appendicitis attended by circumscribed peritonitis the adhesions formed take care of the pus liberated by the sloughing appendix, and the general cavity is protected.

In acute perforating appendicitis with diffuse septic peritonitis, the inflammatory process is a very rapid and destructive one—no barriers of adhesions are formed, perforations suddenly occur, and the whole peritoneum is quickly invaded by septic material.

Sub acute appendicitis usually develops stormily, but tends to run a more or less mild course. It is later attended by perforation, abscess formation, phlebitis, subphrenic abscess, pelvic abscess, abscess of the liver, etc. In these cases the appendix sometimes becomes buried in an inflammatory deposit before perforation occurs, hence, the abscess will for a time be altogether extra-peritoneal. Septic peritonitis may develop later, due to the rupture of the abscess into the peritoneal cavity.

Appendicitis obliterans is superinduced by an attack of acute catarrhal or acute ulcerative appendicitis, and is characterized by a progressive obliteration of the lumen of the appendix, due to the formation and contraction of cicatricial tissue in consequence of the healing of the ulcerations. This process gradually destroys or starves out the glanular tissue of the appendix, and if the ulcerative process is sufficiently extensive, the whole appendix will, in time, become a fibrous cord. Usually these strictures occur near the proximal end of the appendix, but may occur at any point, and not infrequently there are several strictures with saculated spaces intervening. The septic material retained by these sacs finds outlet through the lymphatics, and gives rise to lymphangitis and lymphadenitis, usually of a non-suppurative type. The appendix in these cases may be quite free or surrounded by a localized peritonitis. This variety of appendicitis is said to never form a palpable tumor unless complications develop.

In the chronic recurring appendicitis there are no strictures formed within the appendix, there is simply a chronic catarrhal inflammation that may be associated with some ulceration. This type of the disease may also be rapidly transformed into one of the more dangerous types of appendicitis. There is no involvement of the lymphatics in this variety.

In the diagnosis of appendicitis we can safely depend upon these three constant symptoms: pain, tenderness and rigidity. The pain is paroxysmal, cramp-like and colicky, and for short periods of time may be altogether absent. It is generally referred at first to the umbilicus and above, but after a few hours usually becomes localized in the right iliac fossa; this, however, depends upon the location and direction of the appendix. In a recent case on which I operated the pain was referred to the right kidney and back. At the operation the appendix was found pointing upward and a little inward.

Tenderness is best elicited by gently palpating away from the suspected appendix *first,* then slowly approaching it. A point of extreme tenderness generally means pus formation.

Gentle palpation also makes known to us the presence and degree of rigidity. There are a great many other symptoms of appendicitis, none of which are so constant as these three, and all of us who have had much operative experience in appendicitis know full well that there are no clinical manifestations of the disease by which we can estimate the amount of damage being done, or foretell what changes may occur in a few short hours.

In the differential diagnosis it is no longer necessary to eliminate paratyphlitis, perityphlitis and certain inflammatory lesions of the cæcum, because it has been proven that these are all the outcomes of diseased appendices, and require the same treatment.

The slow onset, headache and the characteristic temperature curve, together with the absence of appendical symptoms, would serve to eliminate typhoid fever. The blood count is also valuable in differentiating the diseases, there being pretty constantly a high leucocytosis in the appendicitis, especially so in cases of pus formation. However, as the appendix is abundantly supplied with lymphoid tissue, it is not surprising that we frequently have symptoms referable to the appendix, if not appendicitis, in typhoid fever.

Typhoid perforations occurring near the appendix sometimes require to be diagnosticated from appendicitis, which is usually done by the history of the case, or in proper cases by an exploratory incision.

In my own practice I have experienced, perhaps, more difficulty in differentiating obstructions of the common bile duct and inflammatory affections of the gall bladder from appendicitis than any other simulating lesion; the history of the case, absence of fever, and the peculiar color and composition of the stools, together with the location and character of the pain, and the absence of the muscular rigidity serves us best in cases of calculi. I have not had a case of appendicitis attended by any amount of peritoneal irritation or inflammation, but that it was also attended by jaundice, and I do not therefore think that jaundice is any more a symptom of liver trouble in doubtful cases than it is of appendicitis.

Cases of intussusception occurring in the region of the appendix are recognized by the sudden appearance of the characteristic tumor and the absence of fever in the first hours of the attack.

Appendicular colic, characterized by sudden localized pain with only slight nausea and vomiting is not accompanied by fever or muscular rigidity and promptly subsides.

The muscular rigidity, the violence of the symptoms, the peculiar character of the pain, the progressive character of the disease, in addition to other symptoms that may be present in the female, will serve to differentiate appendicitis from inflammatory affections of the uterus, adnexa and pelvic cellular tissue. It is to be remembered, however, that a diseased appendix in the female will give rise to some symptoms in consequence of the congestion upon a menstrual epoch.

To foretell the particular variety of appendicitis in a given case is a very difficult problem, and is sometimes beyond the prophetic power of any man, but it is a phase of the subject that should, nevertheless, receive very careful consideration in every case.

Acute catarrhal appendicitis with or without involvement of the wall of the appendix is characterized by the sudden onset of pain, nausea and vomiting, followed by an elevation of temperature and acceleration of the pulse, at the same time there is a rapid formation of a tumor in the right iliac fossa—with this there is tenderness and muscular rigidity over the right side of the abdomen; the rest of the abdomen may be normal. These symptoms, without much change, may persist for some time and subside spontaneously, or promptly pass into another variety of the disease.

Perforation can usually be recognized by a sudden cessation of the characteristic pain and a subsidence of the temperature, attended by symptoms of shock, followed in a short time by symptoms of circumscribed or diffuse peritonitis, this depending upon the amount and location of adhesions formed, and, upon the location and direction of the appendix.

Sub acute appendicitis is recognized by its insidious course, the partial subsidence of the first symptoms and the formation of a circumscribed abscess in the right iliac fossa.

Appendicitis recurrens is recognized by the history of its recurrences and the entire absence of symptoms during the interim.

In appendicitis obliterans (the other chronic variety), owing to the septic material retained (by the strictures) in the appendix, and owing to the infection of the lymphatics by this septic matter, there is never a complete subsidence of all the symptoms, and after a time the patient is rendered an invalid. This is not the case in appendicitis recurrens.

The treatment of appendicitis we will consider under the heads of medical and surgical.

The medical treatment of appendicitis has long ago been characterized by the eminent Professor Deaver of Chicago as "High Grade Christian Science," and I know of no reason yet why he should change his opinion.

In some cases I believe it would be absolutely criminal to think of instituting medical treatment with the hope of relieving the patient, but in selected cases where it is deemed wise to try medical treatment, it should be directed toward putting the patient in that condition in which nature can be depended upon to do the most for him and toward putting him in the best condition for a surgical operation should that become necessary. To accomplish these ends he should be put at absolute rest, in bed on his back, with the thighs slightly flexed on the abdomen. The bowels should be opened with small doses of calomel, oil or salines, and application of ice made to the painful area. No nourishment except small quantities of liquid should be allowed. When the pain is very severe hypodermics of morphine or codeine can be given, but opiates should never be persisted in to the entire relief of the pain; the continuance of the pain should be looked upon as an indication for operation, instead of an indication for more opium. The patient should be carefully and constantly watched and the attendants should hold themselves in readiness to operate upon the first appearance of untoward symptoms. In chronic appendicitis, where operation is refused, or for any reason can not be instituted, medical treatment should be directed, not only toward relieving the attacks, but to their prevention; this will necessitate the education of the patient along these lines, and the regulation of his life and habits.

In my opinion appendicitis in any form is to be regarded as an ultimately fatal disease; that the patient who has had nothing more than appendicæcal colic is carrying about with him a menace to his life; that the patient who has had appendicitis, in ever so light a form one time, is more than likely to have it again, and that each succeeding attack will be more dangerous than the preceding one, and it follows, that I do not believe a patient is cured of appendicitis simply because he has been relieved of its symptoms.

Appendicitis is essentially a surgical disease, and there is no question about whether or not an operation is necessary, but the question of "when to operate," presents itself in every case. In all cases of acute appendicitis with violent symptoms, I believe that the operation is just as much of an emergency as would be an operation for the relief of strangulated hernia.

In cases accompanied by perforation and diffuse peritonitis, an operation is generally useless, but should not be abandoned unless the general condition of the patient will in no wise justify it.

In cases where the peritonitis is circumscribed and the general condition of the patient remains good, I believe that it is safe to delay operation until the abscess can be opened without risk of infecting the general peritonæum.

In recurrent cases when the symptoms are no worse than they have been in previous attacks, I believe that operation can be safely deferred until there is some aggravation of the symptoms, or until the attack has lasted longer than previous attacks without any signs of abatement. I believe that it is as safe to operate on these cases during the attack as it is during the interval, for the reason that if we wait for some complication to develop during the attack to force us into an operation, we wait until it is everlastingly too late. Patients do not generally die of appendicitis, or from the operative interference instituted for its relief, but they do die in consequence of the peritonitis produced by the appendicitis; it is the peritonitis that we hope to prevent by operation—this and nothing more.

Appendicitis obliterans is the only form of appendicitis that in any wise tends to cure itself, and it is so rare that one of the most eminent operators in America, in a series of 5000 operations, has seen less than a dozen cases; however, this exceedingly rare form can be suddenly and rapidly transformed into one of the dangerous types, and promptly end fatally. These cases should always be operated on to prevent future attacks, and to save the patient from a life of invalidism.

Finally, gentlemen, in any case in which we have established a diagnosis of appendicitis and are still unable to foretell the future course of the disease, I believe it is a more hazardous risk to delay than to operate, and that the operation should be promptly performed.

The best working rule with which I am familiar is this of Richardson: "In the early days of severe cases we should operate, first, if the symptoms are becoming more severe (especially if there be any evidence that a sudden distention is taking place); second, if the patient is dangerously septic, shown by pulse and temperature; third, if there is increase in symptoms. In other words, cases that are severe from the first demand an immediate operation, and cases that are mild from the first justify an operation because it is not dangerous.

Hopeless cases are the acute ones already complicated with diffuse septic peritonitis, and those in collapse from perforation or gangrene; these cases are not to be operated on at all, unless they react in such a way as to justify it.

